# Chimeric RNAs Discovered by RNA Sequencing and Their Roles in Cancer and Rare Genetic Diseases

**DOI:** 10.3390/genes13050741

**Published:** 2022-04-22

**Authors:** Yunan Sun, Hui Li

**Affiliations:** 1 Department of Pathology, School of Medicine, University of Virginia, Charlottesville, VA 22908, USA; yushusun10@gmail.com; 2 Department of Biochemistry and Molecular Genetics, School of Medicine, University of Virginia, Charlottesville, VA 22908, USA

**Keywords:** RNA sequencing, chimeric RNA, cis-splicing of adjacent genes, trans-splicing

## Abstract

Chimeric RNAs are transcripts that are generated by gene fusion and intergenic splicing events, thus comprising nucleotide sequences from different parental genes. In the past, Northern blot analysis and RT-PCR were used to detect chimeric RNAs. However, they are low-throughput and can be time-consuming, labor-intensive, and cost-prohibitive. With the development of RNA-seq and transcriptome analyses over the past decade, the number of chimeric RNAs in cancer as well as in rare inherited diseases has dramatically increased. Chimeric RNAs may be potential diagnostic biomarkers when they are specifically expressed in cancerous cells and/or tissues. Some chimeric RNAs can also play a role in cell proliferation and cancer development, acting as tools for cancer prognosis, and revealing new insights into the cell origin of tumors. Due to their abilities to characterize a whole transcriptome with a high sequencing depth and intergenically identify spliced chimeric RNAs produced with the absence of chromosomal rearrangement, RNA sequencing has not only enhanced our ability to diagnose genetic diseases, but also provided us with a deeper understanding of these diseases. Here, we reviewed the mechanisms of chimeric RNA formation and the utility of RNA sequencing for discovering chimeric RNAs in several types of cancer and rare inherited diseases. We also discussed the diagnostic, prognostic, and therapeutic values of chimeric RNAs.

## 1. Introduction

The increasing use of RNA-seq and transcriptomic analysis revealed additional complexities of the transcriptome: (i) diverse non-coding RNAs, for example, pseudogenes, lncRNAs, and circRNAs; (ii) post-transcriptional regulation, which includes alternative splicing, alternative polyadenylation, and RNA editing; (iii) transcribed genetic variants, such as allele-specific expressions and expression-quantitative trait loci [1]; and (iv) chimeric RNAs beyond gene-fusion products. This complexity brings challenges and opportunities to understand the development and progression of human diseases, and thus to discover novel biomarkers and therapeutic targets.

The disease process can be triggered by changes in genes, differences in gene function alone or in combination, lifestyles, and the environment. Genetic disorders include single chromosomal imbalances, gene disorders, epigenetics, and complex disorders [2]. Genetic diseases can be either inherited or acquired. Cancer is an example of an acquired genetic disease [3], as all cancers are genetic at the cellular level, while these genetic changes are often somatic [4]. In recent years, due to the development of RNA-seq, more and more discoveries have been made on the transcriptional level of genetic diseases, including chimeric RNAs, which contain exons from independent parental genes. Chimeric RNAs can be generated through gene fusion, as well as intergenic splicing events, such as long-range intrachromosomal and interchromosomal trans-splicing, as well as cis-splicing between adjacent genes (cis-SAGe) [5]. To date, numerous chimeric RNAs have been found in various cancers and other genetic diseases. They are considered potential diagnostic biomarkers and may be promising therapeutic targets. Yet, recent studies also revealed that chimeric RNA itself is not a cancer or disease-specific phenomenon. Many chimeric RNAs are also uncovered in normal physiology, challenging some traditional views regarding cancer genetics [6,7,8]. Here, we reviewed the formation of chimeric RNAs, focusing mainly on chimeric RNAs that are produced at the RNA level, and their roles in cancer and rare genetic diseases.

## 2. Chimeric RNAs

Historically, many names are interchangeably used in chimeric RNA, such as fusion transcripts, transcription-mediated fusions [9], gene fusions, hybrid genes [10], and tandem chimerism [11]. Here, we use the term “chimeric RNA” to refer to a fusion transcript composed of exons, or fragments of exons from different genes at the RNA level. For the fusion events happening at the DNA level, we prefer the term “gene fusion”.

Chimeric transcripts may provide an alternative means for the tumorigenesis of cancers with a significantly lower mutation burden or maybe a hidden contributor to cancers with multiple carcinogenic sources [5]. Next-generation sequencing technologies enable the efficient identification of genome-wide chimeric transcripts, particularly by long RNA-seq reads [12].

The preservation of the open reading frame altered during chimeric RNA formation could lead to the induction of a novel chimeric protein [13]. Chimeric RNAs could also be candidates serving as competing endogenous RNAs (ceRNA), or microRNA (miRNA) sponges [5]. In addition, by subjecting the whole transcript to nonsense-mediated decay, or swapping 5′/3′ control elements, chimeric RNAs could affect the overall level of parental transcript [13]. Several authors suggested that trans-spliced chimeric RNA may also serve as a template for DNA rearrangement [14,15]. They may also function as scaffolds to bring two genomic loci into close proximity. This RNA–DNA interaction, described as the RNA–poise model, may facilitate genomic translocation [16].

## 3. Formation of Chimeric RNAs

Three main mechanisms that generate chimeric RNAs, frequently observed in cancer, are the gene fusion, trans-splicing, and cis-splicing of adjacent genes.

### 3.1. Cis-Splicing of Adjacent Genes

The cis-SAGe, also called read-through transcript or run-through transcript in the scientific community, transcribes from the DNA sequences of two different adjacent genes that ignore the gene boundary and are spliced into a hybrid mRNA transcript [17]. We prefer the term “cis-SAGe” over “read-through” to avoid the confusion of translational read-through, which describes the skip of a stop codon during protein translation. Additionally, transcriptional read-through has also been used to describe transcripts passing the termination site. We added cis-splicing to emphasize that these chimeras are essentially an alternative splicing between adjacent genes on the same strand. When they are misregulated in cancer, they represent novel cancer biomarkers and/or drug targets [18].

**Figure 1:**
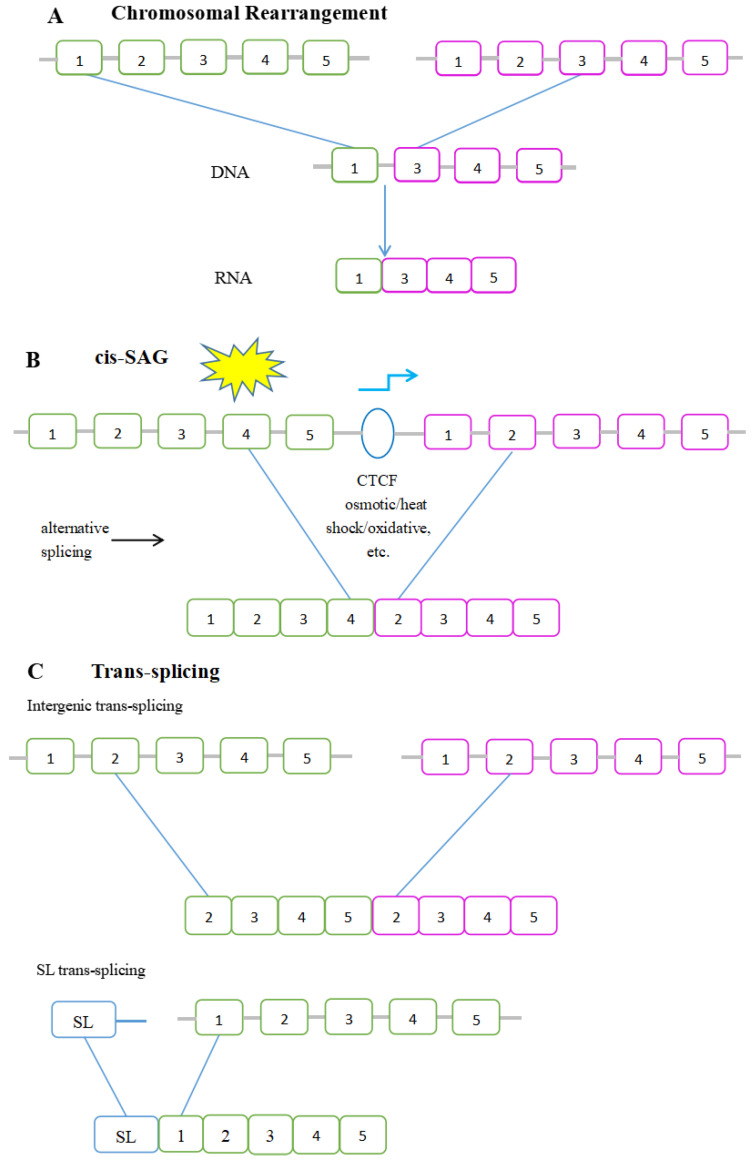
The depiction of chimeric RNAs generated by (**A**) gene fusion; (**B**) cis-SAGe; (**C**) trans-splicing. Bars represent exons and lines represent introns and intergenic regions.

During the formation of cis-SAGe, the transcription program skips the termination signal of the 5′ gene, and the intergenic region is spliced out as an intron to join the exon of the 3′ gene [19]. At least three conditions for cis-SAGe to happen have been proposed: (i) an active primary transcript of the upstream gene; (ii) the ignorance of the gene boundary between two neighboring genes; and (iii) alternative splicing during transcription, as most of the time, the last exon of the 5′ gene and the first exon of the 3′ gene are skipped. In some cis-SAGe chimeric RNAs, specific factors such as the CCCTC binding factor (CTCF), which binds to the insulators between neighboring genes, were shown to affect their production [20,21]. Some studies also showed that stressful conditions such as heat shock, osmotic stress [22], oxidative stress, and infection [23] could have an influence on cis-SAGe [19] (Figure 1).

Many potential cis-SAGe chimeric RNAs have been identified by a systematic in silico analysis and paired-end RNA-seq [5]. Qin et al. analyzed both prostate cancer and non-cancerous samples and observed that 30% of all 300 chimeric RNA events were cis-SAGe chimeras [21]. In another study, 76% of the candidates were classified as cis-SAGe chimeric RNAs [24]. cis-SAGe were also found in healthy tissue samples, suggesting that there is an additional layer of the post-transcriptional control of biological processes that increases the diversity of gene products [17]. Chwalenia et al. developed an efficient and easy cell-based reporter system to study cis-SAGe and identified potential regulators of the process [18].

#### Poly(A) Signal and Transcription Termination in Transcriptional Read Through

Presumably, the skipping of polyadenylation and termination is a prerequisite for cis-SAGe chimeras. However, the exact role that poly(A) addition and transcription termination play in cis-SAGe is still not completely clear.

In the absence of a canonical poly(A)-signal at the 3′ end of the upstream gene, an increase in the read-through caused by the disruption of transcription termination (DoTT) was observed. It was also reported that osmotic stress reduced transcriptional termination of upstream transcripts, which allowed an extension through the downstream of genes (DoG) regions [25]. In a study on the effect of HSV-1 in disrupting transcription termination, the authors reported that HSV-1 infection, salt and heat stress correlate to the read-through transcription extended beyond poly(A) sites, which are derived from DoTT/DoG transcription. The authors further demonstrated that DoTT induced by HSV-1 can lead to increased chromatin accessibility downstream of the affected poly(A) sites [26]. Duc et al. discovered that the loss-of-function in FPA mutants, an Arabidopsis thaliana protein, can promote transcription termination, leading to read-through, and thus the formation of chimeric RNAs [27].

Significant induction of DoGs could be seen at the early time points of osmotic stress, and some DoGs were probably involved in the formation of cis-SAGe chimeras. However, one study reported that different chimeric RNAs responded differently to osmotic stress, which may be due to different choices in distributing primary transcripts to form mature cis-SAGe or stay as read-through [22].

### 3.2. Trans-Splicing

During trans-splicing, a mature mRNA is produced by exons from different RNA transcripts, which are spliced and fused together. RNAs carrying exon repetitions or shuffling exons transcribed from opposite strands can also be generated by intragenic trans-splicing [28]. In addition, chimeric RNAs produced by trans-splicing also include intergenic and spliced leader (SL) trans-splicing. As for intergenic trans-splicing, this results in chimeric mRNA transcripts composed of exons from separate genes. Spliced leader (SL) trans-splicing is a special form of trans-splicing. In this situation, a common SL exon is spliced onto multiple genes [29] (Figure 1).

Trans-splicing is considered a regulatory process to diversify the output of exon-containing genes [30]. Because of their pro-proliferative effects, these events may lead to neoplastic transformation [31]. *JAZF1–JJAZ1* (*SUZ12*) [32] and *PAX3–FOXO1* [33,34] are examples of trans-splicing RNAs. For both chimeric RNAs, the same fusions are generated by different mechanisms in tumors (chromosomal rearrangement) and normal tissues (trans-splicing). These examples also support the idea that trans-spliced RNA may act as a template that facilitates genomic fusion [31].

### 3.3. Gene Fusion

**Figure 2:**
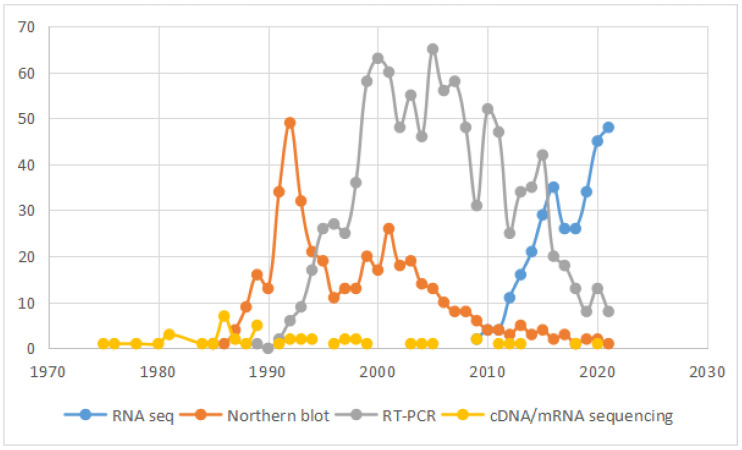
The number of articles using cDNA/mRNA sequencing, Northern blot, RT-PCR and RNA-seq for chimeric RNA detection.

In some literature, the term gene fusion covers both fused DNA as well as RNA level transcripts. Here, by gene fusion, we only refer to DNA-level events to avoid confusion. Gene fusion is generated through a chromosomal rearrangement initiated by DNA double-strand breakage [35]. Subsequently, the fused genes can be transcribed to chimeric mRNAs (Figure 2). Ample studies have been conducted on gene fusions. Many fusion genes have been discovered as biomarkers and therapeutic targets in multiple cancer types.

The most-well-known gene fusion is undoubtedly *BCR-ABL*, generated from t(9;22) in chronic myelogenous leukemia (CML) [36] and in acute lymphoblastic leukemia (ALL) [37]. BCR-ABL inhibitors, such as Imatinib and Dasatinib, are already widely applied in clinical practice [38]. Another well-studied gene fusion is *PAX3-FOXO1* in alveolar rhabdomyosarcoma. It was found to be associated with aggression, metastasis, resistance to chemotherapies, and recurrent and worse prognoses [39]. Treatment indirectly targeting PAX3-FOXO1 by inhibiting its stabilizer Polo-like kinase 1 (PLK1) or its cofactors has been developed [40]. *EML4-ALK* fusion is the most common fusion pattern in ALK-positive lung cancers. Existing medications that are still being improved, such as crizotinib, alectinib and lorlatinib, are used to target ALK rearrangement [41,42,43]. Actionable *FGFR3-F3T3* fusion is found in approximately 3% of gliomas, and there are clinical trials of different FGFR inhibitors (NCT02824133; NCT02052778) with encouraging preliminary results [44]. F3T3 gene fusion independently affects prognosis with a less aggressive clinical evolution and has unique radiogenomic features and a spatial distribution [44]. In primary colorectal carcinoma, *RNF43-SUPT4H1* fusion was found, and the knockdown of its expression had a growth-inhibitory effect on colorectal cancer cells [45]. The fusions reported in endometrial stromal sarcomas (ESS) include *JAZF1-SUZ12*, *YWHAE-FAM22*, *ZC3H7-BCOR*, *MBTD1-CXorf67*, and fusions of *PHF1* with *JAZF1*, *EPC1*, and *MEAF6* [46]. *YWHAE-FAM22A/B* fusion is carried out in histologically high-grade ESS and is clinically more aggressive ESS. *JAZF1-SUZ12* and *MBTD1-CXorf67* fusion are identified in a distinct subgroup of low-grade ESS [47]. *DNAJB1-PRKACA* fusion was found in fibrolamellar hepatocellular carcinoma (FLC) and contributes to tumor pathogenesis [48]. One fusion, *PMP22-ELOVL5*, caused by genomic rearrangements, was discovered in osteosarcoma [49].

Since chimeric RNAs generated by gene fusions in cancer are well known to the scientific community, and the topic has been extensively reviewed, we will mainly focus on chimeric RNAs generated by intergenic splicing in the later cancer section. However, chimeric RNAs in other genetic diseases are relatively new, and many of them have no known generating mechanism. We will discuss them without specifying their categories. Nonetheless, they are all chimeras whose discoveries have benefited significantly from transcriptome sequencing [28].

## 4. RNA Sequencing

Northern blot analysis and RT-PCR were used to detect chimeric RNAs in the past. However, these methods are low-throughput, and can be time-consuming, labor-intensive, and cost-prohibitive, limiting the analyses of chimeric RNAs. Since the year of 2009, the use of RNA sequencing (RNA-seq), an application of next-generation sequencing technology characterizing the whole transcriptome has caused an explosion in the discovery of RNA. In the past twelve years, the number of articles with the keywords “RNA-seq” and “chimeric RNAs” dramatically expanded (Figure 2).

### 4.1. Applications of RNA-Seq in Chimeric RNAs

The three main applications of RNA-seq are the quantification of gene expression, the identification of novel transcripts variants and isoforms, and detection of chimeric RNAs [50]. RNA-seq can also detect multiple alternative splice variants produced by fusion [51]. RNA-seq can reveal the complexity of the dynamic transcriptome and allow for the improved detection of low-abundance transcripts with a higher sequencing depth [52,53]. It has been shown that RNA-seq can provide more actionable clinical hypotheses, and thus is more valuable than whole-exome sequencing (WES) and whole-genome sequencing (WGS) in cancer and Mendelian disorders [54]. Different from the WGS, RNA-seq can be utilized in the detection of fusions generated by intergenic splicing, which only occurs at the RNA level. We believe that RNA-seq will increase the diagnostic rates of genetic diseases, and chimeric RNA detection should be included in RNA-based analytical pipelines [53,55].

The experimental design, choice of library preparation, sequencing depth, and the number of biological replicates all have an influence on the final data output. When analyzing chimeric RNAs, many of which are lowly expressed, a sufficient read depth is required [56]. Moreover, conventional RNA-seq library preparation can cause strand information loss, but this information can be retained using a strand-specific protocol [57].

### 4.2. Computational Methods for Identifying Chimeric RNAs

To date, more than 40 prediction methods have been developed to identify chimeric RNAs from RNA sequencing data [58]. Most bioinformatic prediction tools rely on an initial alignment step. Discordant reads mapping to two different genes are then identified, and a series of filtering and/or realignment steps are applied to identify candidate chimeric RNAs [59]. Some software tools use a genome sequence as a reference, some use transcriptomes, while some others use both. Among them, some methods use k-mer-based matches to decrease the computation time and memory [60]. Criteria used in the filtering steps may include the minimum number of supporting reads (split and spanning reads), the distance between fusion partners, homology-based filters, etc.)

Singh et al. compared 16 chimeric RNA prediction software, including SOAPfuse (BGI Tech Solutions Co., Shenzhen, China), MapSplice (Department of Computer Science, University of Kentucky, Lexington, KY, USA), EricScript (Institute of Biomedical Technologies, University of Florence, Florence, Italy), ChimerScan (National Health Research Institutes, Miaoli Conunty, Taiwan), FusionCatcher (Orion Corporation, Espoo, Finland), JAFFA (Murdoch Children’s Research Institute, Victoria, Australia), TopHat-Fusion (Center for Bioinformatics and Computational Biology, University of Maryland, College Park, MD, USA), pizzly (Department of Molecular and Cell Biology, University of California, Berkeley, CA, USA), FuSeq (Department of Medical Epidemiology and Biostatistics, Karolinska Institutet, Stockholm, Sweden), InFusion (Department of Molecular Biology, Max Planck Institute for Infection Biology, Berlin, Germany), Arriba (German Cancer Research Center DKFZ, Applied Bioinformatics, Heidelberg, Germany), INTEGRATE (McDonnell Genome Institute, Washington University School of Medicine, St. Louis, MO, USA), STAR-Fusion (Broad Institute of MIT and Harvard, Cambridge, MA, USA), STARChip (Department of Genetics and Genomic Sciences, New York, NY, USA), ChimPipe (Centre for Genomic Regulation, The Barcelona Institute of Science and Technology, Barcelona, Spain), and ChimeRScope (Department of Genetics, Cell Biology and Anatomy, University of Nebraska Medical Center, Omaha, NE, USA), for their sensitivity, positive prediction value, F-measure, and computational requirements, and concluded that none of the tools were inclusive since the overlaps of fusions among different software tools were small. Their performance varied depending on the dataset and objects [58]. In general, the performance of individual software methods on simulated datasets is much better than that on real datasets. There are often tradeoffs between sensitivity and the F-measure. JAFFA, SOAPfuse, ChimeraScan, and FuSeq have high sensitivity, and based on F-measure, SOAPfuse or FuSeq will be a better choice [58]. The performances of some tools can be influenced depending on the RNA-seq read length, read number, and the quality of the reads [50]. Different software also have different requirements for time and computational power. In addition to missing true fusion events, they can also produce false positives [61]. Potential artifacts can be generated during reverse transcription, such as RNA 3′ self-priming, 5′ ligation, template switching [62], and sequence similarity [61]. To prioritize the large list of candidate chimeric RNAs, we used multiple filters and discarded those that did not use exon boundaries and low frequencies, focusing on those with biological/clinical implications, such as cancer stage, survival, etc. It was also important to conduct an experimental validation for the predicted chimeras. Popular methods for validation include Northern blotting [63], RNase protection assay [64], nanostring technology [65], and RT-PCR [66,67].

## 5. Chimeric RNAs in Cancer

**Figure 3:**
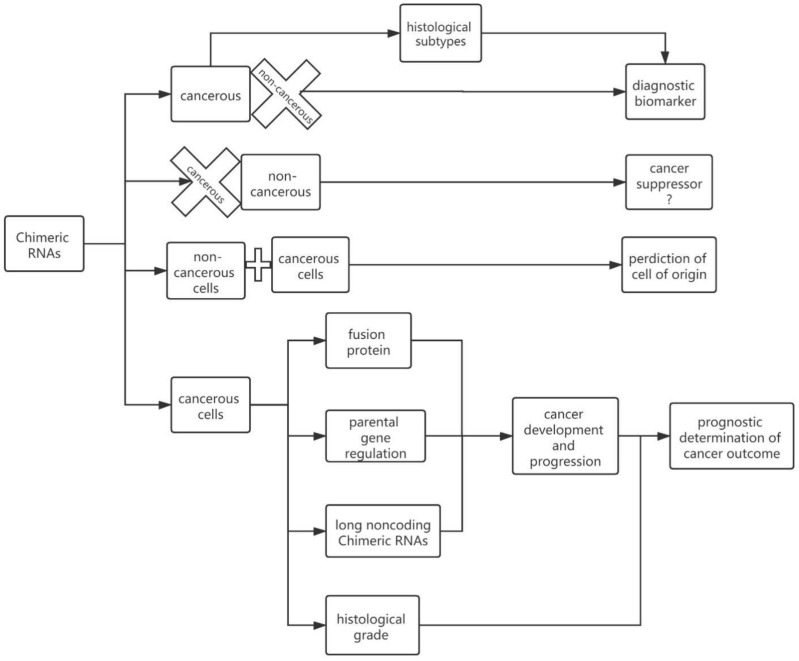
Roles that chimeric RNAs play in cancer. They could act as diagnostic biomarkers, predictors of cells of origin in complicated pathologies, and predictors of prognosis, etc. The crosses represent the absence of chimeric RNAs and the squares represent the presence of chimeric RNAs and their roles.

Chimeric RNAs that were found to be specifically expressed in cancerous cells and/or tissues are potential diagnostic biomarkers. The correlation between chimeric RNAs and histologic differentiation could help to distinguish different subtypes in certain types of cancers. Some chimeric RNAs could also play a role in cell proliferation and cancer development as well as progression, and can be used as tools for cancer prognosis. Chimeric RNAs could participate in gene expression regulation and associate with signal transduction [68]. In some cancers, cell cycle progression can be affected by chimeric RNAs, which could enhance cell growth. The presence of chimeric RNAs in precancer cells, and the fact that the same chimeric RNAs were found in cancerous cells and matched normal cells, could provide insights into the cell of origin for tumors [5] (Figure 3 and Table 1).

### 5.1. Esophageal Cancer

*GOLM1-MAK10* is identified as a highly cancer-enriched chimeric RNA in esophageal squamous cell carcinoma (ESCC). The aberrant chimera, likely derived from cis-SAGe, was associated with histologic differentiation and lymph node metastasis. Furthermore, it encodes a potentially functional secreted fusion protein in human cells, supporting its potential as a clinically useful cancer biomarker that can be detected by standard non-invasive assays [69]. The aberrant level of *ASTN2-PAPPA* antisense chimeric RNA (A-PaschiRNA), which is also likely a cis-SAGe product, is derived from the splicing of exons and the intron antisense of two neighboring genes. It was reported to be associated with tumor progression and patients’ outcomes in human esophageal cancer. In one study, it was confirmed that A-PaschiRNA could be translated into a fusion protein, leading to the discovery that the potential connection between A-Pas fusion protein and the cancer stemness in that A-PaschiRNA could enhance ESCC cancer stemness, facilitating metastases and the cell migration to lymph nodes [70].

### 5.2. NSCLC

Among the 167 analyzed non-small cell lung cancer (NSCLC) cases, *Pe1-Fe3* mRNA was identified in about 10% of the patients without genomic rearrangements, and there was a positive correlation between the presence of chimeric mRNA and the quantities of parental *FER* mRNA. Furthermore, the authors found that *Pe1-Fe3* mRNA-positive patients have significantly shorter progression-free and overall survival periods [71]. With the combined use of RNA-seq, single-molecule RNA FISH, and DNA FISH, Yan et al. detected a cancer sample with *EML4-ALK* chimeric RNA without the *EML4-ALK* gene fusion in an NSCLC cell line. They suggested an RNA–poise model, where spatial proximity of RNA and DNA could poise for the generation of chimeric transcripts. They also demonstrated that RNA-DNA interactions in normal cells were predictive of chimeric transcripts in cancer samples [16]. Maspero et al. identified abundant candidate cis-SAGe transcripts in non-involved lung parenchyma of lung adenocarcinoma patients, which include *CHIA-PIFO*, *CTSC-RAB38*, *ELAVL1-TIMM44*, *FAM162B-ZUFSP*, *IFNAR2-IL10RB*, *INMT-FAM188B*, *KIAA1841-C2ORF74*, *NFATC3-PLA2G15*, *SHANK3-ACR* and *SIRPB1-SIRPD* [72].

### 5.3. Gastric Cancer

A *PPP1R1B-STARD3* chimeric transcript generated by RNA processing was expressed in 21.3% of primary human gastric cancers, while its expression was absent in adjacent-matched normal gastric tissues [73]. In a xenograft mouse model, a PPP1R1B-STARD3 fusion protein promoted significantly larger tumors. The overexpression of *PPP1R1B-STARD3* in MKN-28 significantly promoted cell proliferation and colony formation, and the effect was mediated through phosphatidylinositol-3-kinase (PI3K)/AKT survival pathway [73]. The *DUS4L-BCAP29* chimeric RNA, produced by cis-SAGe, was originally discovered in gastric and prostate cancers [74,75], but later proved to be a ubiquitously expressed chimera in multiple nonneoplastic tissues [76]. Its expression level is similar in noncancerous gastric and prostate cell lines and tissues to those in cancerous ones. Moreover, the overexpression of *DUS4L-BCAP29* promotes cell growth and motility, not only found in cancer but also in normal physiology. These findings highlight the need for caution when nominating a chimeric RNA as a biomarker [77].

### 5.4. Colorectal Cancer

Wu et al. used the Cancer Genome Atlas Colorectal Cancer RNA-seq dataset to conduct extensive data mining on chimeric RNAs and detected that chimeric RNA *RRM2-C2orf48* was frequently expressed in colorectal cancer samples. Moreover, different expression profiles and functions were observed in this chimeric RNA and its parental genes. A high level of expression of *RRM2-C2orf48*, generated by cis-SAGe, correlated with worse outcomes, while the high expression of parental *RRM2* and *C2orf48* was associated with positive clinical outcomes. Cellular proliferation was significantly reduced when *RRM2-C2orf48* was silenced, suggesting that *RRM2-C2orf48* regulated colon cancer cellular proliferation [78].

### 5.5. Tumors of Reproductive System

Li et al. identified the overexpression of cis-SAGe *TSNAX-DISC1* in endometrial carcinoma (EC) [79]. High levels of *TSNAX-DISC1* in EC cells with a stable overexpression of lincRNA-NR_034037 were able to induce G1-S cell cycle progression from the G1 to S phase and promote tumor growth in vivo [79]. Cis-SAGe chimeric RNA *LHX6-NDUFA8* was exclusively detected in cervical cancer tissues and PAP smear samples, but not in normal controls [80].

### 5.6. Prostate Cancer

Kannan et al. reported several chimeric RNAs with a significantly higher incidence in prostate cancer than in matched benign samples, and this incidence was not related to the expression of parental genes. *TMEM79-SMG5* chimera is highly differentially expressed in human cancer samples, indicating its potential as a biomarker [81]. Cis-SAGe-produced chimeric *SLC45A3-ELK4* RNA regulates the cell growth of both androgen-dependent and -independent prostate cancer cells. Its level is associated with disease progression and is the highest in prostate cancer metastasis [82].

Another chimeric transcript specifically detected in prostate cancers but not in normal controls is *D2HGDH-GAL3ST2*, and it is enriched in advanced prostate cancer. Without affecting the parental genes, silencing the chimeric transcript could result in a significant reduction in prostate cancer cell proliferation and motility in both androgen-dependent and castration-resistant cell lines [83].

### 5.7. Renal Cell Carcinoma

A renal cell carcinoma (RCC) study used RNA sequencing and deFuse analysis to analyze cancerous and noncancerous renal cortical tissue samples. Among 68 clear-cell RCCs, 26 chimeric transcripts were identified in 25% (17) of them. In addition, the mRNA expression level of partner genes in cancer samples was significantly correlated with tumor invasiveness and poor prognosis [84]. *CTSC-RAB38* was detected in 20% of clear-cell renal cell carcinoma (ccRCC) samples. Chimeric RNA may also affect the expression of its parental gene. Grosso et al. observed that the increase in *BCL2* mRNA and protein levels was positively correlated with the level of chimeric RNA between *BCL2* and *KDSR* genes located immediately upstream [85].

Another study using next-generation whole transcriptome sequencing as a discovery tool identified a cis-SAGe chimera *TMED6-COG8*, a potential diagnostic marker, in TFE3 translocation renal cell carcinoma (tRCC), since its expression was significantly higher in TFE3 tRCCs compared to clear-cell RCCs and papillary RCC [86]. The expression of another potential diagnostic marker cis-SAGe *BC039389-GATM* in renal cell carcinoma was higher than that in the benign adjacent kidneys; the presence of *KLK4-KRSP1* was observed in 46/169 (27%) RCC, but rarely in normal tissues. *BC039389-GATM* and *KLK4-KRSP1* influence migration and invasion, and *KLK4-KRSP1*-positive patients had shorter overall survival compared to *KLK4-KRSP1*-negative patients. *KLK4-KRSP1* isoform 1 (KKv1) was significantly associated with larger tumors, high-grade tumors, and histological subtypes [87].

### 5.8. Bladder Cancer

Two chimeric RNAs, generated by cis-SAGe, *BCL2L2-PABPN1* and *CHFR-GOLGA3*, were observed to be significantly more expressed in bladder cancer samples in comparison with adjacent normal samples. Furthermore, the authors also suggested that *CHFR-GOLGA3* may play a long noncoding RNA role since it is mainly expressed in the nucleus [88].

Another cis-SAGe chimeric *SYT8-TNNI2* transcript was detected in 37.5% (18/48) of urothelial carcinoma specimens, but not in benign bladder, lung, and breast lesions, indicating it to be a tumor-specific event [89].

### 5.9. Head and Neck Squamous Cell Carcinoma

Cheng et al. found that a chimeric RNA *JMJD7-PLA2G4B* is presented in head and neck squamous cell carcinoma (HNSCC) [90]. *JMJD7-PLA2G4B* promotes HNSCC cell survival by modulating phosphorylation of protein kinase B (AKT). In addition, *JMJD7-PLA2G4B* inhibits the expression of cyclin-dependent kinase inhibitor 1 (p21) and 1B (p27) by regulating an E3 ligase S-phase kinase-associated protein 2 (Skp2), thereby controlling the progression of the cell cycle from the G1 phase to the S phase. Compared with HNSCC cells that only had a JMJD7 knockdown, a knockdown of *JMJD7-PLA2G4B* significantly inhibited the proliferation of HNSCC cells by promoting G1 cell cycle arrest and increased starvation-induced cell death [90].

In patients with nasopharyngeal carcinoma, the low expression of trans-splicing produced *SEPT7P2-PSPH* chimeric RNA induced the expression of downstream gene *PSPH*, promoting cell proliferation and metastasis/invasion, and transforming ability in vitro [91].

The RRM2-c2orf48 expression in the protein level was significantly associated with the T, N, and clinical staging in nasopharyngeal carcinoma patients. Patients with high RRM2-c2orf48 protein expression have a worse disease-free survival rate [92].

### 5.10. Sarcoma

Yuan H et al. discovered that *PAX3-FOXO1* chimeric mRNA was expressed during normal skeletal muscle differentiation (myogenesis) [34]. Further study revealed that numerous chimeric RNAs were transiently expressed during the myogenesis of mesenchymal stem cell (MSC) muscle differentiation. Additionally, all of the chimeric RNAs in RH30, an alveolar rhabdomyosarcoma (ARMS) cell line, could be detected at the myogenic time point of *PAX3-FOXO1* expression, and seven chimeric RNAs followed the exact transient expression pattern as *PAX3-FOXO1*, connecting the disease to a development timepoint. This research suggested that chimeric RNA profiling could be utilized to predict cells of origin in complicated pathologies [93].

In a study of osteosarcoma, *EIF5A-HMGN2* and *EEF1A1-VIM* chimeric transcripts generated by a mechanism independent of gene rearrangements were detected in normal bone and primary osteoblasts by RNA-seq [49].

### 5.11. Others

In seven patients aged 2–14 years with malignant or biologically indeterminate spitzoid tumors, RNA sequencing helped to identify a kinase fusion in five of the six sequenced tumors: *TPM3-NTRK1* in two tumors, complex rearrangement involving *TPM3*, *ALK*, and *IL6R* in one tumor), *BAIAP2L1-BRAF* in one tumor, and *EML4-BRAF* in one disseminating tumor. In addition, two tumors each carried a second fusion gene, *ARID1B-SNX9* or *PTPRZ1-NFAM1* [94]. However, among 17 patients, there was one patient with *CDC5L-BTBD9*, which was generated without structural rearrangement [94].

## 6. Rare Genetic Diseases and Psychological Disorders

Despite the improvement in the diagnostic yield of Mendelian diseases and neuromuscular disorders by gene panels and whole-exome sequencing (WES), a substantial percentage of individuals remain undiagnosed [95]. The potential challenges could be (1) explaining the variants of unknown significance (VUSs); (2) detecting events such as structural rearrangement, copy number variants (CNVs), and tandem-repeat amplification; (3) evaluating gene variations not yet related to disease, and (4) capturing introns and regulatory regions [95]. A number of chimeric RNAs have been revealed in various genetic diseases (Table 2). The RNA-seq approach was reported to increase the genetic diagnostic rate in various rare diseases due to its ability to uncover aberrant expression, aberrant splicing, and mono-allelic expression [53].

Yamada et al. analyzed the RNA-seq data of 56 patients with undiagnosed multiple birth defects using short-read paired-end RNA-seq libraries, and the software ChimPipe was applied. Two patients received a diagnosis through the chimeric analysis. One patient had a chimeric transcript *ZEB2-GTDC1* and was diagnosed with Mowat–Wilson syndrome. *KCNK9-TRAPPC9* chimeric RNA was detected in the second patient who had a diagnosis of Birk–Barel syndrome. Both chimeric RNAs were directly involved in the pathogenesis of the defects [96].

Cousin et al. report a female infant identified as having severe combined immunodeficiencies (NBS SCID) with T cell lymphopenia (TCL). In this case, whole-transcriptome sequencing (WTS) revealed a reciprocal *ATM-SLC35F2* chimeric RNA [97].

Oliver et al. described an inherited-disease-focused workflow of chimeric RNA detection in an undiagnosed, rare-inherited-disease cohort. The study used RNA sequencing and TopHat fusion filtering to detect fusion transcripts in 47 patients with suspected rare inherited diseases [55]. The authors identified 16 fusion candidates passing phenotypic review and after validating by PCR and/or ddPCR, eight candidates were left. In a male child with multiple exostoses, they discovered a *SAMD12-EXT1* fusion resulting from chromosomal deletion, causing the loss of the EXT1 function [55,98]. Another patient was diagnosed with a second fusion *PDPK1-PRSS21,* which could cause Rubinstein–Taybi syndrome. A female infant diagnosed with T cell lymphopenia by newborn screening for severe combined immunodeficiency (SCID) carried a reciprocal *ATM-SLC35F2* fusion. Patients with epilepsy phenotypes, Dyggve–Melchior–Clausen disease, nemaline myopathy, and ZTTK syndrome were found to carry fusions *NARS2-TENM4*, *C18orf32-DYM*, *ARL5A-NEB*, and *SON-FCRL3*, respectively. The *PDPK1-PRSS21* fusion and *SAMD12-EXT1*-carrying patients have unresolved exostoses. With the discovery of *SAMD12-EXT1* and reciprocal *ATM-SLC35F2* fusions, the diagnostic yield is increased by 4.3% [55].

Polymorphic fusion events, *KANSL1-ARL17B* and *TFG-GPR128,* are observed here in various normal tissues, such as lungs, muscles and the skeleton, with greater read support in a large number of patients [99].

### 6.1. Autism

Autism spectrum disorder (ASD) is a group of neurodevelopmental disorders with a high heritability and about 20% of ASDs are syndromic with genetic factors contributing to its pathogenesis. CNVs play an important role in human neuropsychiatric diseases and lead to the generation of chimeric genes, which may increase ASD susceptibility [100].

Some single-nucleotide polymorphisms in the CD38 gene are related to low serum oxytocin levels in ASD patients. Ceroni reported a case with inherited autism, and asthma carried a maternal deletion of 4p15.32 that resulted in a *BST1-CD38* fusion transcript. Although the protein products of BST1 and CD38 were still present, since the reading frame of the *BST1-CD38* fusion transcript was maintained, the level of the expression of both canonical transcripts was reduced [101]. *DOCK4-IMMP2L* fusion transcript caused by the maternally inherited microdeletion encompassing chr7:110,663,978–111,257,682 was found in a patient with ASD and dyslexia [102].

Holt et al. tested 996 individuals with ASD and 1287 controls; they found a duplication involving *EEP1-POLR1A* and a single-occurrence CNV involving *KIAA0319-TDP2*. A validated fusion transcript *MAPKAPK5-ACAD10* was also detected in two probands. However, the fusion transcript had similar rates in both ASD patients and controls and had a premature stop codon; therefore, it is unlikely to influence ASD susceptibility [103].

A study analyzed the genes of a Sardinian family in which two siblings were affected by ASD and their parents were not affected. In both ASD siblings, they detected a rare heterozygous 2p11.2 deletion, consisting of the last coding exons of *ELMOD3*, the entire *CAPG* gene, and the first non-coding exon of *SH2D6*, resulting in a fusion between *ELMOD3* and *SH2D6*. The expression levels of *ELMOD3-SH2D6* gene fusion were significantly higher in subjects carrying the deletion compared to control subjects, suggesting a clinical association with ASD [100].

The translocation mutation of many types of genes, such as transcription factors/regulators, is relevant to ASD. These mutations are listed in a table in a review [104].

### 6.2. Schizophrenia

Rippey et al. screened DNA from 124 affected individuals with schizophrenia. A total of 4 candidate chimeric events were validated in 124 affected individuals and 0 in 290 control individuals. The fusions are *MAP3K3-DDX42*, *DNAJA2-NETO2*, *PLEKHD1-SLC39A9*, and *MATK-ZFR2*. The subcellular localizations of *DNAJA2-NETO2* and *MAP3K3-DDX42* are different from their parent genes. Compared to the *ZFR2* parent gene, *MATK-ZFR2* is likely to be far more highly expressed in the brain during development. These findings indicate that CNVs generated by chimeric genes are a mechanism of schizophrenia [105].

Two fusion transcripts *DISC1-Boymaw* and *Boymaw-DISC1* generated by the DISC1 translocation was found when Zhou et al. re-examined a Scottish schizophrenia family [106,107]. Further research discovered that the expression of the *DISC1-Boymaw* fusion gene inhibits the activity of intracellular NADH oxidoreductase and reduces protein translation, thus damaging brain function and leading to the onset of major mental diseases [108].

### 6.3. Intellectual Disability

Yue et al. reported a case with a developmental delay and macrocephaly that carried a microdeletion with t(7;10)(q33;q23). A fusion gene, *PTEN-SEC8L1*, was produced by the t(7;10) breakpoints, which disrupted genes on both derivative chromosomes [109]. Another de novo translocation t(6;14)(q25.3;q13.2) was described in a male patient with intellectual disability and agenesis of the corpus callosum, leading to fusion transcripts of *MRPP3-ARID1B* [110]. Córdova-Fletes et al. reported a girl with intellectual disability (ID) and neuropsychiatric alterations. A de novo balanced t(10;19)(q22.3;q13.33) translocation was identified in this case, and the translocation led to gene fusions *ZMIZ1-PRR12* and *PRR12-ZMIZ1* [111].

Moyses-Oliveira et al. studied four female patients with intellectual disabilities and found a gene fusion, *ZNF611-IL1RAPL1*, in one of them. It is produced from the der(X) and controlled by the *ZNF611* promoter [112]. There is a report of a 3-year-old boy with an intellectual disability. In the patient’s blood, the authors detected a *GLRB-GRIA2* gene fusion caused by a premature stop codon, and this could be the explanation of the phenotype of the patient [113]. Another study on intellectual disability analyzed three unrelated patients and revealed three chimeric genes, *LIMS1-RANBP2* in patient one, *ARID1B-ZDHHC14* in patient two, and *ZNF451-KIAA1586* in patient three. It is suspected that these fusions are responsible for the phenotypes of patients with intellectual disabilities [114].

## 7. Conclusions and Perspective

Chimeric RNAs can be generated by the RNA trans-splicing and cis-splicing of adjacent genes in addition to chromosomal rearrangement. RNA-seq has a much greater efficiency in detecting chimeric RNAs than other methods such as Northern blot. With the technical development and increasingly widespread utility of RNA-seq, there is an explosive growth of studies about chimeric RNAs. Chimeric RNAs are not specific to cancer since they have been discovered in normal cells, but they may be misregulated in diseases, thus representing novel biomarkers and/or therapeutic targets. Furthermore, chimeric RNA analyses not only improve the diagnostic rate of genetic diseases, including rare inherited diseases as well as cancer, but also provide insights into the mechanism of the initiation and progression of these diseases. In addition, the findings of chimeric RNA benefiting from RNA-seq also enable us to predict the cell of origin for complicated pathologies and the prognosis of cancer.

There are emerging RNA sequencing technologies for studying chimeric RNAs. First, single-cell RNA sequencing (scRNA-seq) enables researchers to study cell heterogeneity and to quantify cell type-specific gene expression in mixed cell populations, facilitating the identification of biomarkers to predict the clinical prognosis and therapeutic targets of cancers. However, it is very rarely if at all used in chimeric RNA detection, due to sequencing depth [115]. Haile et al. introduced a method that produced strand-specific sequencing data, which were ample for identifying cell types and detecting fusion transcripts from heterogeneous biological samples [116]. We envision that with the increase in sequencing depth and the advance in single-molecule sequencing, scRNA-seq will play a promising role in detecting chimeric RNAs. Second, spatial transcriptomics helps researchers gain deeper insight into biology, extending from simple tissues to more complex structures. In particular, spatial transcriptomics can study the molecular characteristics at the boundary between cancer and normal tissues [117]. This can be applied to detect fusion transcripts in cancer cell lines and clinical tissue data. For instance, the recently developed STfusion and C-scores can be used to spatially localize fusion transcripts in clinical tissue sections at an almost single-cell level [17].

## Figures and Tables

**Table 1:** The roles of chimeric RNAs generated by different mechanisms in different types of cancer and normal cells.

	Type of Cell/Tissue		Chimeric RNAs	Formation	Function
Cancer	Esophageal Cancer		*GOLM1-MAK10*	cis-SAGe	correlates with histologic differentiation; lymph node metastasis; encodes a secreted fusion protein
			*ASTN2-PAPPA*	splicing of exons and intron antisense of two neighboring genes	aggravates tumor progression and metastasis
	NSCLC		*Pe1-Fe3*	alteration at the transcriptome level (trans-splicing)	correlates with poor postoperative survival periods
			*EML4-ALK*	Chromosomal rearrangement/trans-splicing	promotes NSCLC tumorigenesis
	Gastric Cancer		*PPP1R1B-STARD3*	cis-SAGe	promotes tumorigenesis through activation of PI3K/AKT signaling
			*DUS4L-BCAP29*	cis-SAGe	promotes cell growth and motility
	Colorectal Cancer		*RRM2-C2orf48*	cis-SAGe	promotes cell proliferation and correlates with poor clinical outcomes
	Tumors of Reproductive System	EC	*TSNAX-DISC1*	cis-SAGe	induces G1-S cell cycle progression and enhance cell growth
		cervical cancer tissues	*LHX6-NDUFA8*	cis-SAGe	Diagnostic biomarker
	Prostate Cancer		*TMEM79-SMG5*	splicing	Diagnostic biomarker
			*SLC45A3-ELK4*	cis-SAGe	correlates with disease progression and metastases
			*D2HGDH-GAL3ST2*	cis-SAGe	Promotes cell proliferation and migration
	Renal Cell Carcinoma		*CTSC-RAB38*	cis-SAGe	
			*TMED6-COG8*	cis-SAGe	Diagnostic biomarker
			*BC039389-GATM*	cis-SAGe	Diagnostic biomarker
			*KLK4-KRSP1*	cis-SAGe	Diagnostic biomarker; associates with worse clinical outcome, larger tumors, high grade tumors, the histological subtype
	Bladder Cancer		*BCL2L2-PABPN1*	cis-SAGe	Diagnostic biomarker
			*CHFR-GOLGA3*	cis-SAGe	Diagnostic biomarker
			*SYT8-TNNI2*	cis-SAGe	Diagnostic biomarker
	HNSCC		*JMJD7-PLA2G4B*	cis-SAGe	promotes cells proliferation by inhibiting cell cycle arrest in G1 phase; controls AKT phosphorylation to promote SCC cell survival
	nasopharyngeal carcinoma		*SEPT7P2-PSPH*	trans-splicing	promote cell proliferation and metastasis/invasion by up-regulating the expression of the downstream gene *PSPH*
	osteosarcoma		*EIF5A-HMGN2*	trans-splicing	
			*EEF1A1-VIM*	trans-splicing	
	spitzoid tumors		*CDC5L-BTBD9*	no structural rearrangement	
Non-cancer	diverse non-cancerous cell lines (mammary gland, lung epithelial, and foreskin fibroblast, etc.)		*DUS4L-BCAP2*	cis-SAGe	promotes cell growth and motility
	endometrial stromal cells		*JAZF1-SUZ12*	trans-splicing	increases cell proliferation
	normal skeletal muscle differentiation (myogenesis)		*PAX3-FOXO1*	trans-splicing	interferes with the muscle differentiation process; contributes to tumorigenesis
	normal bone and primary osteoblasts		*EIF5A-HMGN2 EEF1A1-VIM*	trans-splicing	
	non-involved lung tissue of lung adenocarcinoma		*CHIA-PIFO*	cis-SAGe	plays a functional role in asthma and possibly other lung inflammatory conditions
			*CTSC-RAB38*	cis-SAGe	Maintains lung surfactant homeostasis and lamellar body morphology
			*ELAVL1-TIMM44*	cis-SAGe	involves in lung cancer cell apoptosis
			*NFATC3-PLA2G15*	cis-SAGe	epithelial–mesenchymal transition (EMT)
			*IFNAR2-IL10RB*	cis-SAGe	
			*KIAA1841-C2ORF74*	cis-SAGe	Relates to ciliated epithelial cells
			*SHANK3-ACR*	cis-SAGe	Involves in cell growth, angiogenesis and epithelial–mesenchymal transition
			*SIRPB1-SIRPD*	cis-SAGe	

**Table 2:** Chimeric RNAs in other types of genetic diseases and psychological diseases.

Diagnosis		Chimeric RNA
Birth defects	Mowat–Wilson syndrome	*ZEB2-GTDC1*
	Birk–Barel syndrome	*KCNK9-TRAPPC9*
	Immunodeficiencies (NBS SCID) with T cell lymphopenia (TCL)	*ATM-SLC35F2*
		*SAMD12-EXT1*
	Rubinstein–Taybi syndrome	*PDPK1-PRSS21*
	epilepsy phenotype	*NARS2-TENM4*
	Dyggve–Melchior–Clausen disease	*C18orf32-DYM*
	Nemaline myopathy	*ARL5A-NEB*
	ZTTK syndrome	*SON-FCRL3*
	Unresolved	*PDPK1-PRSS21*
	Unresolved	*SAMD12-EXT1*
Autism		*BST1-CD38*
		*DOCK4-IMMP2L*
		*EEP1-POLR1A*
		*KIAA0319-TDP2*
		*MAPKAPK5-ACAD10*
		*ELMOD3-SH2D6*
Schizophrenia		*MAP3K3-DDX42*
		*DNAJA2-NETO2*
		*PLEKHD1-SLC39A9*
		*MATK-ZFR2*
		*DISC1-Boymaw* and *Boymaw-DISC1*
Intellectual disability		*PTEN-SEC8L1*
		*MRPP3-ARID1B*
		*PRR12-ZMIZ1*
		*ZNF611-IL1RAPL1*
		*GLRB--GRIA2*
		*LIMS1-RANBP2*
		*ARID1B-ZDHHC14*
		*ZNF451-KIAA1586*
